# Case Report: Exertional rhabdomyolysis in a spin class participant with sickle cell trait

**DOI:** 10.12688/f1000research.16326.2

**Published:** 2019-07-01

**Authors:** Teresa Longo, Matthew Shaines

**Affiliations:** 1 Albert Einstein College of Medicine, Bronx, NY, 10461, USA; 2 Department of Internal Medicine, Montefiore Medical Center, Bronx, NY, 10467, USA

**Keywords:** Rhabdomyolysis, Spin class, Sickle cell trait

## Abstract

Exertional rhabdomyolysis is more common in sickle trait due to a predisposition to dehydration and inability to concentrate the urine. Spinning, an indoor cycling workout, has been associated with exertional rhabdomyolysis in recent reports. A consequence of rhabdomyolysis is acute kidney injury, which may be expected to be more common in patients with sickle trait. We report a case of spinning induced rhabdomyolysis in a woman with sickle trait that did not result in renal injury. “Spin rhabdo” is thought to be more severe than other causes of exertional rhabdomyolysis and is associated with higher creatine kinase levels than other causes of exertional rhabdomyolysis. Therefore, individuals with known sickle trait should consider visiting their physician prior to participation in spin classes for the first time. We might also consider voluntary screening for sickle trait in at risk populations prior to enrolling in spin classes given that many patients are unaware of their sickle trait status.

## Introduction

Rhabdomyolysis is a clinical syndrome consisting of intense muscle breakdown and necrosis. It is caused by a variety of triggers, including trauma, infection, drugs, and intense exertion. Nontraumatic exertional rhabdomyolysis is more common in patients with disorders of glucose and lipid metabolism or sickle cell disease, although it may occur in individuals with normal muscles if exertion is significant enough to impair muscle oxygenation. Spinning is a high intensity stationary cycling exercise performed in a group setting and often synchronized to music. It has recently been linked to case reports of exertional rhabdomyolysis in otherwise young, healthy individuals ^1^.

One of the major complications of rhabdomyolysis is acute kidney injury (AKI), caused by a combination of volume depletion and accumulation of myoglobin pigment released by damaged muscle cells inside the renal tubules. Measurement of creatine kinase (CK) levels in the serum is an important indicator of muscle necrosis ^2^; a recent study found that CK levels are higher in spinning associated rhabdomyolysis than in other causes of exertional rhabdomyolysis ^3^. This finding implies the degree of muscle necrosis may be higher in spinning induced rhabdomyolysis, a correlation that is especially concerning for individuals predisposed to renal injury. Sickle cell trait (SCT), while usually considered benign, may predispose patients to renal problems such as hematuria and hyposthenuria. Here, we report a case of “spin class rhabdomyolysis” treated with aggressive intravenous hydration without acute kidney injury in a young woman with sickle cell trait.

## Case report

A 28-year-old Hispanic female with no significant medical history presented to the emergency department with acute onset bilateral leg pain and dark urine 3 days after attending a spin class for the first time. She did not regularly participate in high intensity workouts, although she did frequently walk for exercise. The class consisted of 45 minutes of high intensity cycling. She did not drink much water before or during the class. Several hours after the class, her legs felt weak and gave out. She endorsed bilateral thigh pain that she described as heavy, sore, and limiting her activity. One day prior to admission she noticed that her urine was brown in color, which prompted her to present to the emergency department.

She never experienced similar symptoms in the past. She denied sick contacts, recent travel, recent illness, fevers or feeling overheated, significant alcohol or drug use, or history of deep vein thrombosis. She took no medications and had no allergies. Although she had one episode of pyelonephritis several years prior to presentation, she reported no personal or family history of renal disorders. Family history was unremarkable for known genetic disorders, musculoskeletal disorders, or metabolic myopathies.

Vital signs were stable upon presentation. Her thighs were tender to palpation and strength of lower extremities was limited by pain. Dorsalis pedis pulses were palpable but weak. There was large blood on urine dipstick and minimal red blood cells on urine microscopic analysis. Creatine kinase (CK) was 74,978 IU/L and she was admitted with a diagnosis of rhabdomyolysis. Renal function was normal. She was treated with aggressive intravenous isotonic saline and sodium bicarbonate. Upon discharge, CK was decreasing and she was able to tolerate large intake of oral fluids. A hemoglobin electrophoresis pattern was performed at outpatient follow up to search for a potential underlying cause of her rhabdomyolysis. The result was consistent with sickle cell trait: HbA 57.8% and HbS 37.6%.

## Discussion

Many factors can predispose to rhabdomyolysis, including trauma, metabolic disorders, sickle cell disease, dehydration, high temperature, medications, and excessive exercise ^4^. There has been an increase in the number of admitted patients with exertional rhabdomyolysis in recent years ^5^. While sickle cell trait is usually considered benign, a 2016 study found a significant association between sickle trait and exertional rhabdomyolysis in U.S Army soldiers ^6^. Sickle trait may predispose to dehydration due to an inability to concentrate urine and conserve water in conditions of strenuous exercise, which leads to lactic acid buildup and erythrocyte sickling ^7^. The etiology of rhabdomyolysis in our patient was likely multifactorial, and may be attributable to inadequate hydration and lack of regular exercise prior to participation in the spin class. The presence of sickle cell trait may have predisposed her to rhabdomyolysis in the setting of these factors. However, we must acknowledge that SCT is common, and despite its high prevalence most people with SCT do not develop rhabdomyolysis. Therefore, it possible her SCT was merely an incidental finding and she developed rhabdomyolysis due entirely to other factors, namely poor hydration in the setting of high-intensity exercise.

Spinning is a high intensity, indoor bicycle sport often synchronized to music that has been growing in popularity. It has been associated with exertional rhabdomyolysis most commonly in young, otherwise healthy females. The majority of cases occur in women under the age of 35 years ^8^. While cases of “spin rhabdo” occur overwhelmingly in first time spin class participants with deconditioning, patients often have no other predisposing factors. Rhabdomyolysis may occur after as little as 15 minutes of spinning ^9^. Spectrum of illness is variable and ranges from mild to severe with potential for life threatening complications such as AKI and compartment syndrome ^1^. One study suggested that patients with spinning induced rhabdomyolysis showed more severe disease and longer length of hospital admission than patients with rhabdomyolysis of other causes ^8^. Additionally, a 2016 retrospective cohort study found that spinning induced rhabdomyolysis was associated with higher CK levels than other causes of exertional rhabdomyolysis ^3^.

The risk of AKI is thought to be lower when CK values are less than 15-20,000 IU/L on admission ^10^. Multiple studies have shown a weak correlation between serum CK value and incidence of AKI, and renal injury has occurred at CK levels as low as 5,000 IU/L ^10^. This correlation has one major implication, namely that individuals with sickle cell trait should be especially cautious when participating in spin classes for the first time. The higher CK levels associated with spinning induced rhabdomyolysis may be particularly concerning for individuals with risk factors for kidney disease, such as those with sickle trait. Therefore, we recommend that individuals with known sickle trait visit a physician prior to participation in spin classes and discuss ways to minimize their risk of rhabdomyolysis.

In many parts of the world, newborn screening to detect both sickle cell disease and trait is widely available. In the United States, all states are now mandated to offer screening; while most parents opt to have their children tested, reporting of the result to families is variable. In a 2006 survey, only 16% of respondents were aware of their sickle trait status ^11^. A number of sports organizations advocate for universal screening prior to participation in high intensity athletics due to rare reported instances of exercise related sudden death ^12^. In 2010, the National Collegiate Athletic Association (NCAA) implemented a mandatory opt out sickle trait screening policy prior to athletic participation ^13^. However, screening in adults remains controversial due to the potential for loss of employment, insurance, and other forms of discrimination based on sickle trait status. This risk must be weighed against the potential benefits from screening, which include the opportunity for providers to discuss preventative measures with patients. Given the severity of spinning-induced rhabdomyolysis and its increasing incidence in recent years, we may consider voluntary screening for sickle trait in at risk populations prior to enrolling in spin classes for the first time. However, as evidence of an association between SCT and exertional rhabdomyolysis at the time of this article’s publication is limited mainly to case reports, further studies are needed before a strong, evidence-based recommendation can be made. Nevertheless, this case warrants further investigation into whether or not extremely elevated CK values are more prevalent in patients with SCT. The point that SCT is highly prevalent and the vast majority of SCT individuals do not develop rhabdomyolysis is an important one. There is also no way of knowing how many patients with rhabdomyolysis have SCT because they are seldom screened. This case also highlights the importance that individuals seek medical care prior to starting a high intensity exercise regimen for the first time; those thought to be at increased risk for exertional rhabdomyolysis should be educated on prevention.

## Conclusion

Given the longer length of hospital admission and higher CK values associated with spinning induced rhabdomyolysis, individuals at high risk of exertional rhabdomyolysis should consider talking to their primary care physician before participating in spin classes. Prevention of exertional rhabdomyolysis may require more aggressive hydration to maintain fluid and electrolyte balance in individuals with sickle trait than in normal spin class participants, although more research is needed to clarify the association between SCT and exertional rhabdomyolysis. Organizers of spin classes should take precautions to ensure novel participants hydrate well and do not overexert themselves during the class. Voluntary screening for sickle trait in at risk populations prior to enrolling in spin classes may be considered given many individuals are unaware of their sickle trait status.

## Consent

Written informed consent for publication of their clinical details and/or clinical images was obtained from the patient.

## Data availability

No data is associated with this article.

## References

[1] Ramme AJ Vira S Alaia MJ: Exertional rhabdomyolysis after spinning: case series and review of the literature. *J Sports Med Phys Fitness.* 2016;56(6):789–93. 25665750

[2] Baird MF Graham SM Baker JS: Creatine-kinase- and exercise-related muscle damage implications for muscle performance and recovery. *J Nutr Metab.* 2012;2012: 960363. 10.1155/2012/960363 22288008 PMC3263635

[3] Cutler TS DeFilippis EM Unterbrink ME: Increasing Incidence and Unique Clinical Characteristics of Spinning-Induced Rhabdomyolysis. *Clin J Sport Med.* 2016;26(5):429–31. 10.1097/JSM.0000000000000281 27604073

[4] Bosch X Poch E Grau JM: Rhabdomyolysis and acute kidney injury. *N Engl J Med.* 2009;361(1):62–72. 10.1056/NEJMra0801327 19571284

[5] Aalborg C Rød-Larsen C Leiro I: An increase in the number of admitted patients with exercise-induced rhabdomyolysis. *Tidsskr Nor Legeforen.* 2016;136(18):1532–6. 10.4045/tidsskr.15.1207 27731596

[6] Nelson DA Deuster PA Carter R 3rd: Sickle Cell Trait, Rhabdomyolysis, and Mortality among U.S. Army Soldiers. *N Engl J Med.* 2016;375(5):435–442. 10.1056/NEJMoa1516257 27518662 PMC5026312

[7] Dincer HE Raza T: Compartment syndrome and fatal rhabdomyolysis in sickle cell trait. *WMJ.* 2005;104(6):67–71. 16218320

[8] Kim YH Ham YR Na KR: Spinning: an arising cause of rhabdomyolysis in young females. *Intern Med J.* 2016;46(9):1062–8. 10.1111/imj.13168 27346188

[9] Brogan M Ledesma R Coffino A: Freebie Rhabdomyolysis: A Public Health Concern. Spin Class-Induced Rhabdomyolysis. *Am J Med.* 2017;130(4):484–487. 10.1016/j.amjmed.2016.11.004 27908792

[10] Kenny JE: Creatine Kinase: How Much is Too Much? *The NYU Langone Online Journal of Medicine.* 2010 Reference Source

[11] Treadwell MJ McClough L Vichinsky E: Using qualitative and quantitative strategies to evaluate knowledge and perceptions about sickle cell disease and sickle cell trait. *J Natl Med Assoc.* 2006;98(5):704–710. 16749645 PMC2569269

[12] Blinder MA Russel S: Exertional sickling: questions and controversy. *Hematol Rep.* 2014;6(4):5502. 10.4081/hr.2014.5502 25568759 PMC4274478

[13] McDonald MA Creary MS Powell J: Perspectives and Practices of Athletic Trainers and Team Physicians Implementing the 2010 NCAA Sickle Cell Trait Screening Policy. *J Genet Couns.* 2017;26(6):1292–1300. 10.1007/s10897-017-0107-6 28578465

