# Supplementary material for: Case Report: Exertional rhabdomyolysis in a spin class participant with sickle cell trait
Source: F1000Res. 2019 Jul 1;7:1742. Originally published 2018 Nov 2. [Version 2] doi: 10.12688/f1000research.16326.2 (PMC6659762; doi:10.12688/f1000research.16326.2)
Supplement: Peer review history

# Peer review: Reviewer response for version 2

**DOI:** 10.5256/f1000research.21239.r50612

**Published:** 2019-07-24

**Authors:** Nyamkhishig Sambuughin

**Affiliations:** 1 The Henry M. Jackson Foundation for the Advancement of Military Medicine, Bethesda, Maryland, USA; 2 Consortium for Health and Military Performance, Department of Military and Emergency Medicine, Uniformed Services University of Health Sciences, Bethesda, USA

Authors revised this case report to reflect the complexity of rhabdomyolysis and its variable causes. As stated in the report, the development of rhabdomyolysis in the studied case is likely due to a combination of factors, being unaccustomed to intense exercise (i.e. spinning), dehydration and Sickle Cell Trait.

I confirm that I have read this submission and believe that I have an appropriate level of expertise to confirm that it is of an acceptable scientific standard.

# Peer review: Reviewer response for version 2

**DOI:** 10.5256/f1000research.21239.r50613

**Published:** 2019-07-16

**Authors:** Gregory Kato

**Affiliations:** 1 Pittsburgh Heart, Lung, Blood and Vascular Medicine Institute, University of Pittsburgh, Pittsburgh, PA, USA

The authors have addressed my prior comments adequately in this revised version. The points made are valid and the relevant issues are addressed in a balanced manner.

I confirm that I have read this submission and believe that I have an appropriate level of expertise to confirm that it is of an acceptable scientific standard.

# Peer review: Reviewer response for version 1

**DOI:** 10.5256/f1000research.17835.r45794

**Published:** 2019-04-02

**Authors:** Nyamkhishig Sambuughin

**Affiliations:** 1 The Henry M. Jackson Foundation for the Advancement of Military Medicine, Bethesda, Maryland, USA; 2 Consortium for Health and Military Performance, Department of Military and Emergency Medicine, Uniformed Services University of Health Sciences, Bethesda, USA

Individuals with Sickle Cell Trait (SCT), generally a benign carrier state of hemoglobin mutation, are thought to be at increased risk for exertional rhabdomyolysis. This report presents an association between SCT and rhabdomyolysis triggered by spinning. The association between SCT and exertional rhabdmyosis is mostly based on case reports. There are no well-designed case-control study that address this association except epidemiological study in US army soldiers. In many case reports, including this one, authors often highlight SCT is a main contributing risk factor. However, etiology of rhabdomyolysis is extremely variable. Although this case report describes an event of rhabdomyolysis in sufficient details, other potential contributing factors were not well discussed. The subject in the report appears to be unaccustomed to intense exercise and was not adequately hydrated before and during exercise. These are well known factors that contribute to rhabdomyolysis. Combination of those factors will certainly increase subjects' risk to muscle necrosis. Individuals should be aware of these factors before enrolling in unaccustomed strenuous exercise, regardless if they have SCT or not.

I confirm that I have read this submission and believe that I have an appropriate level of expertise to confirm that it is of an acceptable scientific standard, however I have significant reservations, as outlined above.

# Peer review: Reviewer response for version 1

**DOI:** 10.5256/f1000research.17835.r44105

**Published:** 2019-02-25

**Authors:** Gregory Kato

**Affiliations:** 1 Pittsburgh Heart, Lung, Blood and Vascular Medicine Institute, University of Pittsburgh, Pittsburgh, PA, USA

This a fairly balanced presentation of a case report of a case of exertion-related rhabdomyolysis and its potential association with sickle cell trait (SCT). There is suggestive evidence for association of exertion-related rhabdomyolysis with SCT, but only with assumed prevalence of SCT in military personnel of self-identified African descent. There has never been a well-controlled analysis of this sickle cell risk, but it is strongly suspicious based on extensive circumstantial evidence. There also is an extensive history in the 1960's of stigma and discrimination involving SCT that is not well-appreciated in the current generation of biomedical personnel.

 The authors do a good job of presenting the issues involved in this case report with the exception of three points:

1. At least half of the cases of exertional rhabdomyolysis do not involve SCT, and so SCT is not a complete explanation of risk. Universal precautions help to address this population outside of SCT.
2. SCT is highly prevalent and the vast majority of SCT subjects never develop rhabdomyolysis, including in spin classes, and additional risk factors must contribute to these rare cases.
3. There are inadequate data to base evidence-based recommendations and more research is needed in this area.

I confirm that I have read this submission and believe that I have an appropriate level of expertise to confirm that it is of an acceptable scientific standard, however I have significant reservations, as outlined above.
